# cAMP‐phosphodiesterase 4D7 (PDE4D7) forms a cAMP signalosome complex with DHX9 and is implicated in prostate cancer progression

**DOI:** 10.1002/1878-0261.13572

**Published:** 2024-01-05

**Authors:** Chloe Gulliver, Tara Busiau, Ashleigh Byrne, Jane E. Findlay, Ralf Hoffmann, George S. Baillie

**Affiliations:** ^1^ School of Cardiovascular and Metabolic Health, College of Medical, Veterinary and Life Science University of Glasgow UK; ^2^ Oncology Solutions Philips Research Europe Eindhoven The Netherlands

**Keywords:** DHX9, disruption, PDE4D7, phosphorylation, proliferation, prostate cancer

## Abstract

A robust body of work has demonstrated that a reduction in cAMP‐specific 3′,5′‐cyclic phosphodiesterase 4D isoform 7 (PDE4D7) is linked with negative prostate cancer outcomes; however, the exact molecular mechanism that underpins this relationship is unknown. Epigenetic profiling has shown that the *PDE4D* gene can be hyper‐methylated in transmembrane serine protease 2 (*TMPRSS2*)–ETS transcriptional regulator ERG (*ERG*) gene‐fusion‐positive prostate cancer (PCa) tumours, and this inhibits messenger RNA (mRNA) expression, leading to a paucity of cellular PDE4D7 protein. In an attempt to understand how the resulting aberrant cAMP signalling drives PCa growth, we immunopurified PDE4D7 and identified binding proteins by mass spectrometry. We used peptide array technology and proximity ligation assay to confirm binding between PDE4D7 and ATP‐dependent RNA helicase A (DHX9), and in the design of a novel cell‐permeable disruptor peptide that mimics the DHX9‐binding region on PDE4D7. We discovered that PDE4D7 forms a signalling complex with the DExD/H‐box RNA helicase DHX9. Importantly, disruption of the PDE4D7–DHX9 complex reduced proliferation of LNCaP cells, suggesting the complex is pro‐tumorigenic. Additionally, we have identified a novel protein kinase A (PKA) phosphorylation site on DHX9 that is regulated by PDE4D7 association. In summary, we report the existence of a newly identified PDE4D7–DHX9 signalling complex that may be crucial in PCa pathogenesis and could represent a potential therapeutic target.

## Graphical abstract

Decreased expression of PDE4D7 is associated with poor prognosis and aggressive prostate cancer (PCa). Here, we report an interaction between PDE4D7 and DHX9 which is implicated in PCa growth, as well as the identification of a PKA phosphorylation site on DHX9. It is speculated that PDE4D7 modulates cAMP signalling around DHX9, mediating its phosphorylation by PKA.

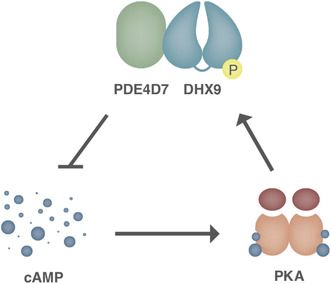

**Abbreviations**
AI: androgen insensitive
AR: androgen receptor
AS: androgen sensitive
cAMP: cyclic adenosine monophosphate
CRPC: castration‐resistant prostate cancer
DHX9: DExH‐Box helicase 9
PCa: prostate cancer
PDE4D7: phosphodiesterase type 4D7
PPI: protein–protein interaction
UCR1/2: upstream conserved region 1/2

## 1 Introduction

In the UK, prostate cancer (PCa) accounts for the most common cancer among men, causing approximately 12 000 deaths each year [1]. Phosphodiesterase type 4 (PDE4s) are responsible for the degradation of cyclic AMP (cAMP) in order to modulate and compartmentalise cAMP signalling around specific signalling complexes [2, 3]. The PDE4 family are of relevance in diseases such as cancer, with the PDE4D sub‐family in particular implicated in PCa development and progression [4, 5]. There are nine isoforms within the human PDE4D sub‐family (PDE4D1–9) which can be characterised into long, short or super‐short in accordance with which conserved regulatory regions they contain [6]. PDE4D7 is a long isoform containing both upstream conserved region 1 (UCR1) and UCR2 domains and localises to the sub‐plasma membrane in PCa cells [5]. The expression of PDE4D7 is significantly diminished during progression from androgen sensitive (AS) localised PCa to castration resistance PCa (CRPC), implicating it in the development of an aggressive disease phenotype [5]. As a consequence, measuring transcript level of *PDE4D7* has shown promising prognostic value in evaluating longitudinal outcomes in patients with PCa, lending itself as an important biomarker of disease progression and recurrence [5, 7, 8, 9]. Aside from the clear potential of PDE4D7 as a prognostic biomarker, these studies also reveal a protective role for PDE4D7 in PCa patients. Protein–protein interactions (PPIs) allow formation of signalling networks through the tethering of enzymes, such as PDEs, to protein complexes [10]. It is likely that PDE4D7 influences PCa signalling through its interaction with other proteins in cAMP sensitive signalosomes. Identification of PPIs in the PCa context would boost understanding of the intracellular networks driving PCa tumourigenesis, as well aiding therapeutic development for disrupting or enhancing PPIs as a treatment of PCa [11].

DExD/H‐box RNA helicases function in the unwinding and remodelling of genomic structures in order to regulate cellular processes such as transcription, translation and replication, to promote maintenance of genomic stability [12, 13]. Whilst structurally similar due to their conserved DExD/H motif II amino acid signature sequence [14], individual DExD/H‐box RNA helicases can have highly specific functions. In cancer, DExH‐Box helicase 9 (DHX9) has been of much interest lately as the enzyme is involved in the unwinding of both single‐ and double‐stranded nucleic structures, particularly DNA/RNA G‐quadruplexes and RNA displacement loops (R‐loops). Given its fundamental roles in maintaining genomic integrity, dysregulation of DHX9 function can have profound consequences in promoting malignant development [15]. DHX9 can have conflicting roles in cancer progression by acting as both an oncogene or a tumour suppressor, depending on its interacting partners and compartmentalised connected signalling pathways [15]. Overall, overexpression of DHX9 is a common feature of many tumours, including colorectal cancer [16], lung cancer [17] and hepatocellular carcinoma [18]. With regards to prostate cancer the relationship is less clear, however DHX9 maps to chromosome 1q25 in humans, corresponding to the major prostate cancer susceptibility locus [19]. Additionally, a recent report highlighted a regulatory influence of DHX9 over androgen receptor (*AR*) transcription [20]. DHX9 also lies downstream of transcription factor *SOX4*, a proposed oncogene frequently overexpressed in PCa, delineating *DHX9* as a putative SOX4 transcriptional target [21].

Interestingly, PDE4D7 and DHX9 show opposing expression patterns in PCa; whilst DHX9 upregulated expression is positively correlated to PCa progression from AS to androgen insensitive (AI) disease [20], PDE4D7 expression is inversely correlated [8, 9]. The molecular mechanisms that manifest PDE4D7's influence over PCa growth are still unknown; therefore, we sought to investigate potential signalling networks in which PDE4D7 has a role in PCa cells. Here, we present for the first time, the identification of a direct interaction between PDE4D7 and DHX9. Using peptide array technology, we have successfully mapped the PDE4D7‐DHX9 binding domain, as well as identifying a putative PKA phosphorylation site on DHX9. Furthermore, through the development of a cell‐penetrating peptide to disrupt the PDE4D7‐DHX9 PPI, real‐time growth analysis revealed disruption of this complex suppressed neoplastic growth of PCa cells, highlighting the complex's involvement in PCa pathogenesis.

## 2 Materials and methods

### 2.1 Cell culture and treatments

LNCaP clone FGC (ATCC: CRL‐1740™, RRID: CVCL_1379) WT and stable shRNA‐PDE4D7 knockdown [generated by AMSBIO (Abingdon, UK)] cell lines were cultured in RPMI 1640 medium (Gibco™, Paisley, Scotland), supplemented with 10% (v/v) foetal bovine serum, 1% (v/v) _L_‐Glutamine and 1% (v/v) penicillin/streptomycin. DU145 (ATCC: HTB‐81™, RRID: CVCL_0105), VCaP (ATCC: CRL‐2876, RRID: CVCL_2235) and HEK293 (ATCC: CRL‐1573, RRID: CVCL_0063) cell lines were cultured in Dulbecco's modified Eagle's medium (Sigma‐Aldrich, Burlington, MA, USA) supplemented with 10% (v/v) foetal bovine serum, 1% (v/v) _L_‐Glutamine and 1% (v/v) penicillin/streptomycin. VCaP media was further supplemented with 1 mm sodium pyruvate. All cell lines were authenticated and certified by ATCC (Gaithersburg, MD, USA) and routinely checked for mycoplasma contamination. Cells were cultured within a 37 °C incubator with 5% CO_2_.

Cells were treated with the following compounds reconstituted in DMSO: 25 μm forskolin (Sigma, F6886), 100 μm IBMX (Sigma, I5879), 10 μm rolipram (Sigma, R6520), 10 μm UCR1‐disruptor peptide, 10 μm scrambled peptide.

### 2.2 DNA constructs and transfection

Plasmid DNA constructs were transiently transfected into cell lines using Lipofectamine LTX with Plus Reagent (ThermoFisher Scientific, Waltham, MA, USA) according to manufacturer's protocol. DNA plasmids constructs used were pcDNA3.1‐PDE4D7‐VSV, pcMV3‐DHX9‐FLAG, pcMV3‐DHX9‐S449A‐FLAG, pcMV3‐DHX9‐S449D‐FLAG, pGEX‐5X‐1 4D UCR1‐GST (Dr G Bolger, University of South Alabama), pGEX‐4X‐1 GST (Dr Y Sin, University of Glasgow). Site‐directed mutagenesis of pcMV3‐DHX9‐Flag plasmid to insert point mutations S449A and S449D using In‐Fusion HD Cloning Plus kit (Takara Bio, Shiga, Japan) as per manufacturer's guidelines. Bacterial transformation and colony selection was performed prior to purification using QIAprep Spin Miniprep Kit (Qiagen, Hilden, Germany).

### 2.3 xCELLigence Real‐Time cell analysis (RTCA)

Real‐time cell growth was analysed using the xCELLigence RTCA system (Agilent, Santa Clara, CA, USA), which measures cell adhesion across microelectrode biosensors on 96 well E‐plates within a 37 °C incubator with 5% CO_2_. Cellular adhesion influences electrical impedance which is converted into the cell index (CI) via rtca software. Initial background measurements were recorded with medium only, followed by addition of 10 000 cells per wells for measurement of CI over time. Approximately 24 h after seeding, cells were treated with specified concentrations of compounds. Negative controls containing either 0.1% DMSO or untreated cells were also included. CI was transformed to normalised CI via the rtca software to timepoint of treatment.

### 2.4 Peptide array

Peptide arrays were synthesised in‐house on cellulose membrane supports via automatic SPOT synthesis using the MultiPep RSi Robot (Intavis, Tübingen, Germany) and 9‐fluorenylmethoxycarbonyl chloride (Fmoc) chemistry. Peptide sequences from DHX9 and PDE4D7 were spotted as 25‐mer peptides shifted by five amino acids, alongside alanine scans, N‐ and C‐terminal truncations and point substitutions to identify crucial amino acids for protein–protein interactions. For identification of PKA phosphorylation of DHX9, peptide arrays were incubated with or without 100 units of active bovine PKA catalytic subunit. For binding between DHX9 and PDE4D7, membranes were overlaid with either purified protein or overexpressing cell lysate. Far western blotting was then conducted with the following antibodies prior to chemiluminescent imaging: anti‐GST 1 : 1000 (Santa Cruz Biotechnology, Dallas, TX, USA, #sc‐138), anti‐phospho‐PKA‐substrate 1 : 1000 (Cell Signalling, Danvers, MA, USA, #9624S), anti‐FLAG 1 : 1000 (ThermoFisher, #PA1‐984B), Goat‐anti‐rabbit HRP 1 : 2000 (Jackson ImmunoResearch, #111‐035‐144), Rabbit Anti Mouse HRP 1 : 2000 (Jackson ImmunoResearch, West Grove, PA, USA, 315‐035‐003).

### 2.5 Immunoprecipitation + mass spectrometry

#### 2.5.1 Immunoprecipitation

Immunoprecipitation was carried out with 500 μg cell lysate for endogenous PDE4D7 or DHX9, and overexpressed DHX9‐FLAG or PDE4D7‐VSV cell lysate incubated overnight in Protein G Sepharose beads (Invitrogen, Waltham, MA, USA) with 1 μg·μL^−1^ of the appropriate antibody or IgG control; anti‐DHX9 (Abcam, #ab26271), anti‐PDE4D7 (Baillie Lab, University of Glasgow, Glasgow, UK), anti‐VSV (Abcam, Cambridge, UK, #ab1874), anti‐FLAG (Sigma‐Aldrich, #F3165), mouse IgG (Millipore, Burlington, MA, USA, NI03), rabbit IgG (Millipore NI01), sheep IgG (ThermoFisher #31243). Samples were centrifuged four times at 500 × ***g*** for 3 min prior to elution of bound protein complexes in 2 × Laemmli buffer for 5 min at 95 °C. Samples were analysed via SDS/PAGE and immunoblotting, then imaged via LI‐COR Odyssey (Lincoln, NE, USA).

#### 2.5.2 Mass spectrometry

HEK293 lysate overexpressing PDE4D7‐VSV or control vector were incubated with VSV‐agarose beads (or FLAG‐agarose beads as a control), and immunoprecipitated as described above. Samples were subjected to SDS/PAGE prior to Coomassie staining of gels. Bands representing PDE4D7 pulled down proteins were excised alongside corresponding bands in control sample lanes and analysed via mass spectrometry.

### 2.6 Subcellular fractionation

Subcellular fractionation was performed as per manufacturer's instructions using the Qproteome® Cell Compartment Kit (Qiagen) to isolate protein lysate from membrane, cytoplasmic and nuclear compartments.

### 2.7 Western blotting

Cells were lysed in 3T3 lysis buffer (50 mm NaCl, 50 mm NaF, 30 mm sodium pyrophosphate, 25 mm HEPES, 2.5 mm EDTA, 10% (v/v) glycerol, 1% (v/v) Triton X‐100; pH 7.5) supplemented with cOmplete™ EDTA‐free protease inhibitor cocktail (Sigma‐Aldrich). Protein concentrations were determined via Bradford assay. Equal concentrations of protein samples were separated via SDS–polyacrylamide gel electrophoresis (SDS/PAGE) and transferred onto nitrocellulose membranes for immunoblotting. Membranes were blocked in 5% (w/v) milk powder or 5% bovine serum albumin (w/v) in TBS‐T for 1 h prior to primary and secondary antibody incubations in the same solution. Primary antibodies used: anti‐PDE4D7 1 : 500 (Baillie Lab), anti‐VSV 1 : 5000 (Abcam, #ab1874), anti‐GAPDH 1 : 5000 (Abcam, #ab8245), anti‐DHX9 1 : 2000 (Abcam, #ab26271), anti‐FLAG 1 : 2000 (Abcam, #ab1257), anti‐phospho‐DHX9 1 : 200 (Baillie Lab), anti‐phospho‐PKA substrates 1 : 1000 (Cell Signalling, #9624S), anti‐GM130 1 : 1000 (Abcam, #ab52649), anti‐HSP90 1 : 1000 (Santa Cruz Biotechnology, #sc‐7947). Secondary antibodies used: IRDye® 800CW Donkey‐anti‐Rabbit IgG 1 : 5000 (LI‐COR, #926‐32213), IRDye® 680RD Donkey‐anti‐Goat IgG 1 : 5000 (LI‐COR, #926‐68074), IRDye 800CW Goat‐anti‐Human IgG 1 : 5000 (LI‐COR, #926‐32232), IRDye® 680RD Donkey‐anti‐Mouse IgG 1 : 5000 (LI‐COR, #925‐68072).

### 2.8 RT‐qPCR

RNA was extracted using the RNeasy Mini Kit (Qiagen) and one‐step qRT‐PCR was performed with the Superscript™ III Platinum™ One‐Step qRT‐PCR Kit (ThermoFisher Scientific) as per manufacturers' protocols. Experiments were carried out using the Step‐One™ Real‐Time PCR System (ThermoFisher Scientific) at the following parameters: One cycle of 50°C for 30 min and 95°C for 5 min, followed by 45 cycles of 95°C for 15 s and 60°C for 45 s. Primers and probes were purchased from Integrated DNA Technologies (Coralville, IA, USA) (Table 1), with 400 nm primer/200 nm probe and 1 ng·μL^−1^ RNA per reaction. *C*_t_ values were quantified using the $2^{-\Delta \Delta C_{t}}$ method and statistical analysis was performed on ∆∆*C*_t_ values.

**Table 1:** Primers and probe sequences used for RT‐qPCR. PUM1, TBP, ACTB and HPRT1 were used as housekeeping genes. All probes were labelled 5′ 6‐FAM/ZEN/3′ IBFQ.

Gene	Accession No.	Forward primer (5′–3′)	Reverse primer (5′–3′)	Probe (5′–3′)
PDE4D5	NM_001197218.1	GCTTCTCAGCAGCAACATC	TGCCATTGTCCACATCAAAA	ACAGCGGCGTTTCACGGTGGCACA
PDE4D7	NM_001165899.1	GAACATTCAACGACCAACCA	TGCCATTGTCCACATCAAAA	CTGCCGCTGATTGCTATCACTTCTGCA
PDE4D9	NM_001197220.1	ATGAGCATTATTATGAAGCCAAGATC	GTGCCATTGTCCACATCAAAAC	CTACAAGTTCCCTAAGGACTGCAGAGG
DHX9	NM_001357.5	TGTACTGTAGGTGTGCTCCT	CAACATCACGCAGTACTACCA	TGACTGATTCCTCGAATGCCTGCTTC
PUM1	NM_001020658.2	GCCAGCTTGTCTTCAATGAAAT	CAAAGCCAGCTTCTGTTCAAG	ATCCACCATGAGTTGGTAGGCAGC
TBP	NM_003194.4	GCCAAGAAGAAAGTGAACATCAT	ATAGGGATTCCGGGAGTCAT	TCAGAACAACAGCCTGCCACCTTA
HPRT1	NM_000194.2	GAGGATTTGGAAAGGGTGTTTATT	ACAGAGGGCTACAATGTGATG	ACGTCTTGCTCGAGATGTGATGAAGG
TUBA1B	NM_006082.3	TGACTCCTTCAACACCTTCTTC	TGCCAGTGCGAACTTCAT	CCGGGCTGTGTTTGTAGACTTGGA

### 2.9 Immunocytochemistry

Cells were seeded onto glass coverslips 24 h prior to transfection. Cells were fixed with 4% (v/v) paraformaldehyde, blocked and permeabilised with 10% donkey serum, 0.5% BSA and 0.2% Triton‐X in PBS. Coverslips were incubated overnight with specified antibodies; Anti‐PDE4D7 1 : 200 (Baillie Lab), anti‐DHX9 1 : 500 (Abcam, #ab26271), anti‐NDH‐II 1 : 1000 (Santa Cruz Biotechnology, #137232), anti‐phospho‐DHX9 1 : 100 (Baillie Lab), anti‐FLAG 1 : 500 (Sigma‐Aldrich, #F2555), anti‐VSV 1 : 500 (Abcam, #ab1874), anti‐phospho‐PKA‐substrate 1 : 1000 (Cell Signalling, #9624S), Wheat Germ Agglutinin (ThermoFisher Scientific, #W11261) Alexa‐Fluor anti‐sheep 488 1 : 500 (ThermoFisher Scientific, #A‐11055), Alexa‐Fluor anti‐rabbit 546 1 : 500 (ThermoFisher Scientific, #A10040), Alexa‐Fluor anti‐mouse 546 1 : 500 (ThermoFisher Scientific, # A10036). Coverslips were mounted onto slides with Duolink *In Situ* Mounting Medium with DAPI (Sigma‐Aldrich) and imaged via the ZEISS (Jena, Germany) LSM 880 laser scanning microscope with 63× oil immersion objective.

### 2.10 Proximity ligation assay

Cells were seeded and fixed as previously described, prior to incubation with 1 mg·mL^−1^ wheat germ agglutinin Alexa‐Fluor 488 (ThermoFisher Scientific, #W11261). Cells were then permeabilised with 0.1% Triton‐X in PBS for 10 min. PLA was performed using the Duolink® *In Situ* Red Starter Kit Goat/Rabbit (Sigma‐Aldrich, #DUO92105) as per manufacturer's protocol, with antibodies as stated in immunocytochemistry methods. Z‐stack images were performed via the ZEISS LSM 880 laser scanning microscope with 63× oil immersion objective.

### 2.11 Statistical analysis

Values are presented as mean ± standard error of mean (SEM) for *N* ≥ 3, unless otherwise stated. Statistical analyses were performed either via one‐way ANOVA + Dunnett's multiple comparison, two‐way ANOVA + Šidák's multiple comparison, or via unpaired *t*‐test with assumed Gaussian distribution. *P* ≤ 0.05 was deemed significant (* = *P* ≤ 0.05, ** = *P* ≤ 0.01, *** = *P* ≤ 0.001, **** = *P* ≤ 0.0001). All statistical analyses and models were performed via graphpad prism 9 (San Diego, CA, USA).

## 3 Results

### 3.1 Identification of PDE4D7 and DHX9 as binding partners

DU145 and PC3 are the most widely used PCa cell lines to represent AI PCa as they no longer express the AR, whilst LNCaP and VCaP cells are models for AS PCa, with VCaP retaining AR expression and LNCaP harbouring a mutated AR [22]. The expression of PDE4D7 and DHX9 show an inverse correlation between AI and AS PCa cells in terms of both mRNA (Fig. S1A) and protein (Fig. S1B), whereby PDE4D7 expression decreases during androgen insensitivity and disease progression, whereas DHX9 expression increases. Interestingly, whilst low PDE4D7 is correlated with an androgen insensitive phenotype, treatment of LNCaP cells with enzalutamide (AR antagonist) did not lead to changes in PDE4D7 expression (Fig. S2). DU145 cells represent an important negative control in experiments due to their lack of PDE4D7 expression, whilst the retained PDE4D7 expression in LNCaP and VCaP alongside DHX9 justify their use in characterising the PDE4D7‐DHX9 complex in PCa.

**Fig. 1:**
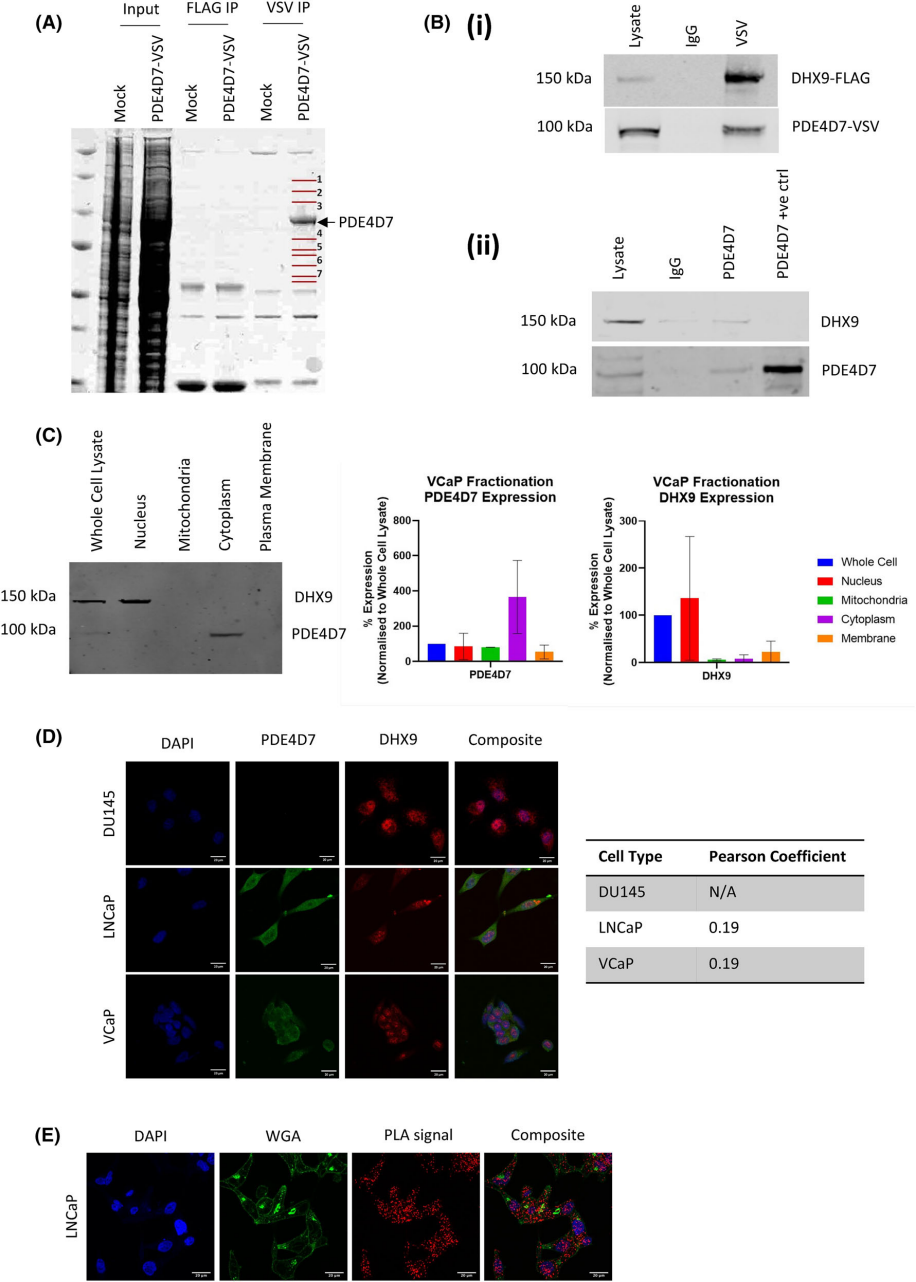
Identification of complex formation between PDE4D7 and DHX9. (A) SDS/PAGE following immunoprecipitation of PDE4D7‐VSV‐transfected HEK293 cells with VSV‐ or FLAG‐conjugated agarose beads. Inputs are total cell lysate. Bands represented by red bands 1–7 were excised and analysed via mass spectrometry, alongside PDE4D7‐corresponding band (*N* = 3). (B) (i) Immunoprecipitation of PDE4D7‐VSV from overexpressing HEK293 cells, probed for FLAG‐tagged DHX9 and VSV‐tagged PDE4D7. (ii) Immunoprecipitation of endogenous PDE4D7 in VCaP cells, membrane probed with PDE4D7 and DHX9 antibodies. Data are representative of *N* = 3 experiments (IgG – immunoglobulin G, +ve ctrl – positive control). (C) Subcellular fractionation and SDS/PAGE of VCaP cells. Quantification of PDE4D7 and DHX9 in each cellular compartment (mean ± SEM for *N* = 2). (D) PDE4D7 and DHX9 expression via immunocytochemistry in DU145, LNCaP and VCaP cells. Pearson's coefficient is presented as mean ± SEM for *N* = 15 cells per cell line, for *N* = 3 independent experiments. Scale bars measure 20 μm. (E) Interaction between PDE4D7 and DHX9 via proximity ligation assay (PLA) in LNCaP cells. Representative of five images per replicate for *N* = 3, scale bars measure 20 μm. PLA signal – red, wheat germ agglutinin (WGA) – green, DAPI – blue.

In order to discover novel signalling pathways that may have a role in PCa we attempted to interrogate the PDE4D7 interactome. Identification of PDE4D7 PPIs was determined by immunoprecipitation (IP) coupled with mass spectrometry. PDE4D7‐VSV was immunoprecipitated from transiently transfected HEK293 cells using VSV‐agarose beads followed by SDS/PAGE. Seven specific protein bands that co‐immunoprecipitated with PDE4D7‐VSV were excised (Fig. 1A) and analysed by mass spectrometry alongside corresponding bands of the same molecular weight from mock VSV IP for comparison. Four proteins were identified in the PDE4D7‐VSV IP sample, namely PDE4D7, heat shock cognate 71 kDa protein (HSPA8), octamer‐binding protein (NONO) and ATP‐dependant RNA helicase A (DHX9). As DHX9 is transcribed from a gene that maps to the prostate cancer susceptibility locus and has been implicated in numerous cancer types [15], we selected this candidate for further investigation.

Firstly, to further prove existence of the complex, HEK293 cells were co‐transfected with PDE4D7‐VSV and DHX9‐FLAG followed by IP with VSV‐agarose beads (Fig. 1B (i)). Both PDE4D7‐VSV and DHX9‐FLAG were found in the VSV IP, confirming that the transfected proteins can be found in the same complex. Additionally, endogenous PDE4D7 was immunoprecipitated from VCaP PCa cells using a PDE4D7‐specific antibody, and we found that endogenous DHX9 co‐immunoprecipitated with the purified PDE4D7 (Fig. 1B (ii)). Given the evidence of interaction between PDE4D7 and DHX9, we sought to determine the cellular locations of both proteins in PCa cell lines. Subcellular fractionation of VCaP cells revealed differences in cellular distribution between the two proteins, with PDE4D7 found mostly in the cytoplasm and membrane whereas DHX9 was predominantly located in the nucleus (Fig. 1C). To further confirm locations, androgen sensitive cell lines (VCaP and LNCaP) and the androgen insensitive cell line, DU145, were immuno‐stained for PDE4D7 and DHX9 and imaged via confocal microscopy. As previously reported, DU145 cells did not express PDE4D7 and served as a negative control resulting in a Pearson coefficient of 0, whereas in VCaP and LNCaP cells PDE4D7 could be seen throughout the cell (Fig. 1D). DHX9 expression was observed in all cell lines, localised predominantly in the nucleus. The Pearson coefficient of the whole cell between PDE4D7 and DHX9 was 0.19 in both LNCaP and VCaP cells, suggesting little overlap in localisation between these proteins *in vitro*. As both proteins appeared to have different cellular locations, proximity ligation assay (PLA) was performed in LNCaP cells to determine whether we could detect PDE4D7‐DHX9 complexes in PCa cells (Fig. 1E). Clusters of PDE4D7‐DHX9 interactions (red dots) could be observed predominantly in the nucleus but also in the cytoplasm of both cell lines. A positive PLA signal indicates that the two proteins are within 40 nm from one another [23], suggesting that despite having largely differing distributions there was enough overlap to allow formation of signalling complexes containing PDE4D7 and DHX9.

### 3.2 Mapping the binding domains between PDE4D7 and DHX9

**Fig. 2:**
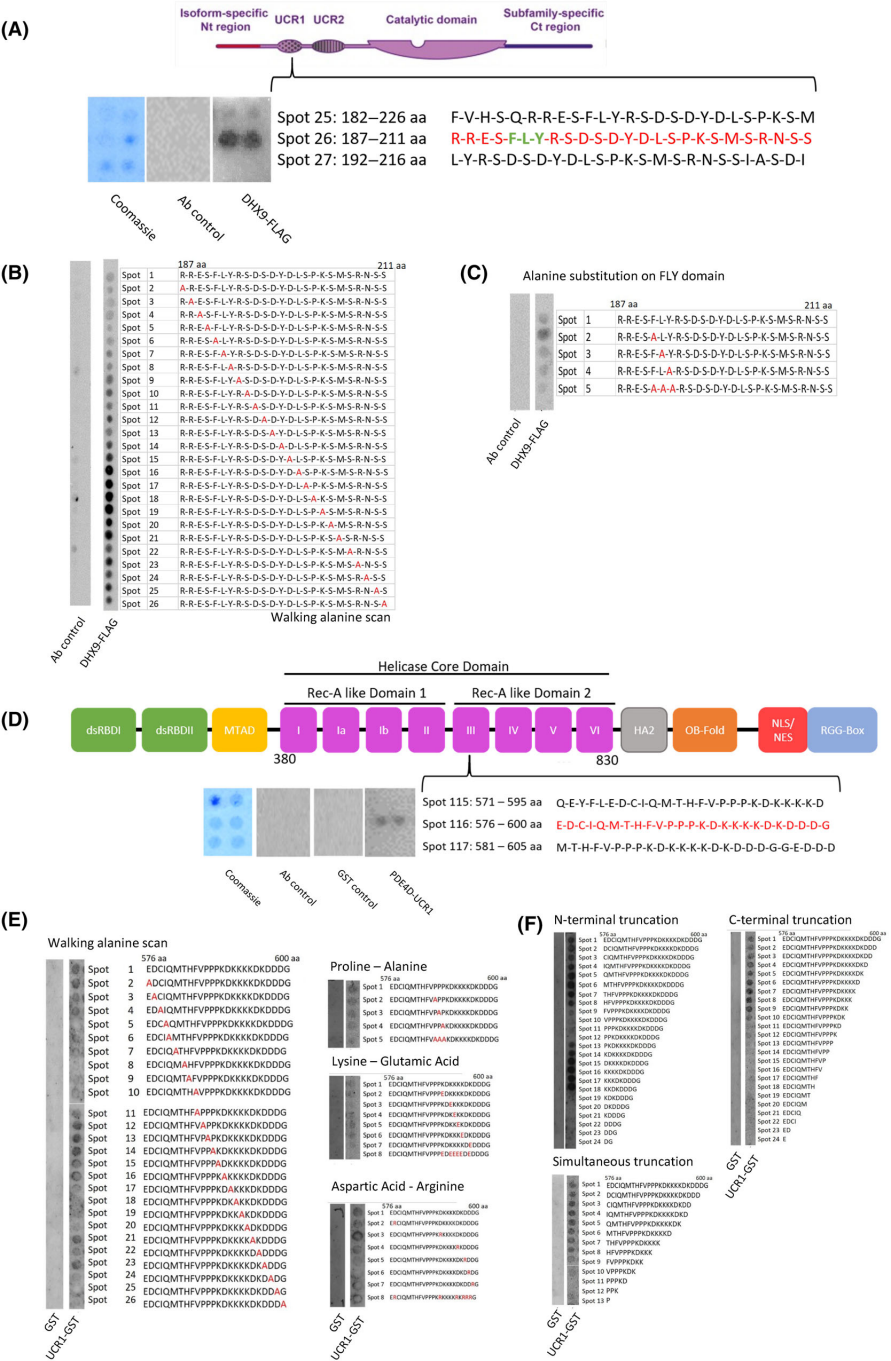
Mapping putative binding site between PDE4D7 and DHX9. (A) Peptide array of full‐length PDE4D7 sequence overlaid with DHX9‐FLAG overexpressing HEK293 lysate and incubated with FLAG antibody. Identified DHX9 binding site sequence in red. Protein structure diagram adapted from Tibbo et al. [50] under the CCBY 4.0 licence. Walking alanine scan (B) and alanine substitutions of FLY docking site (C) of 25mer DHX9 binding sequence. (D) Peptide array of full‐length DHX9 sequence overlaid with purified GST‐tagged PDE4D‐UCR1 and incubated with GST antibody. Identified PDE4D7 binding site sequence in red. Walking alanine scan and amino acid substitutions (E) and N‐, C‐ and dual N + C‐terminal truncations (F). All figures are representative of *N* = 2 experiments. aa, amino acid; Ab, antibody; Ct, C‐terminal; Nt, N‐terminal; UCR1/2, upstream conserved region 1/2.

To map the binding sites between PDE4D7 and DHX9, we constructed immobilised peptide array libraries of overlapping 25mers (each sequentially shifted by 5 amino acids) that encompassed the entire PDE4D7 sequence. The PDE4D7 peptide library was overlain with lysate from HEK293 cells overexpressing DHX9‐FLAG, and a far‐western blot for the FLAG tag undertaken to determine whether any of the PDE4D7 peptides would bind to the helicase. Interestingly, a peptide corresponding to a sequence in the UCR1 region of PDE4D7 showed robust binding (indicated by dark spots) to DHX9‐FLAG (Fig. 2A). Alanine scanning of the UCR1 25mer sequence did not identify any single amino acids as being essential (Fig. 2B) however triple alanine substitution of the ‘FLY’ motif severely attenuated binding (Fig. 2C). This motif is part of a recently discovered multi‐functional docking domain for PDE4 binding partners [24].

Next, to define the PDE4 binding site on DHX9 we performed a reciprocal experiment where we overlayed purified PDE4D‐UCR1‐GST recombinant protein (Fig. S3) onto a full‐length DHX9 peptide array (25mers shifted by 5 amino acids) and probed for the GST tag. This time we detected a binding site for PDE4‐UCR1 within the Rec‐A like Domain 2 of the DHX9 helicase core domain (Fig. 2D). Walking alanine scans and point substitutions of the identified 25mer sequence (Fig. 2E) identified lysine‐alanine substitutions between spots 18–20 that decreased binding, as well as lysine‐glutamic acid substitutions that inhibited the interaction. Follow‐up N‐terminal truncations, C‐terminal truncations, and simultaneous N‐ and C‐truncations (Fig. 2F) reveal loss of binding upon removal of F^584^ – P^587^ N‐terminus region, as well as removal of K^591^. Overall, our analysis identified a core binding domain of ‘HFVPPPKDK’ which seemed to be essential for UCR1 association.

### 3.3 Identification of a novel PKA‐phosphorylation site on DHX9

**Fig. 3:**
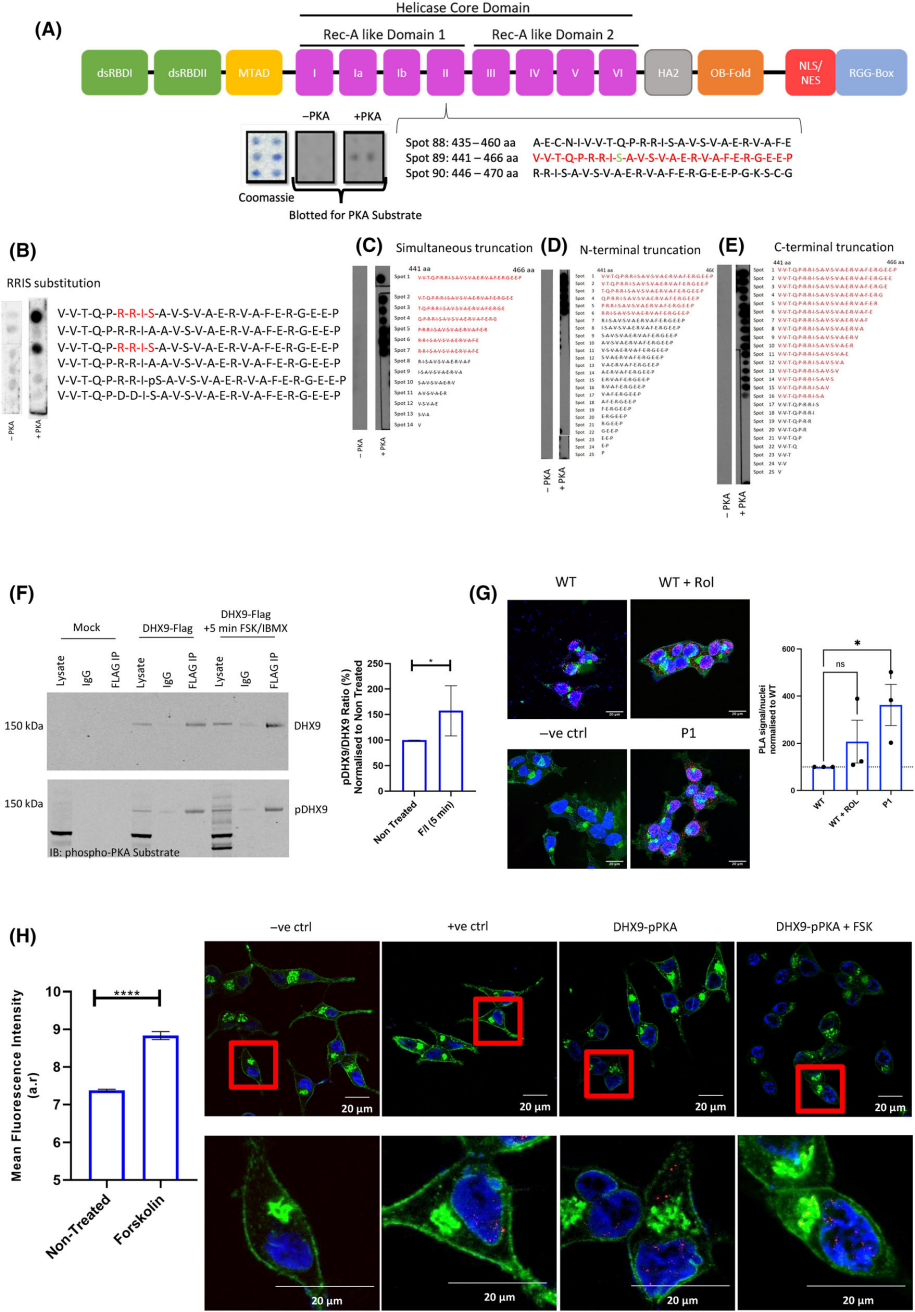
Identification of novel PKA‐phosphorylation site on DHX9. Peptide array of full‐length DHX9 sequence overlaid with purified recombinant PKA catalytic subunit and ATP, and incubated with phospho‐(Ser/Thr) PKA substrate antibody (A). Peptide array of identified PKA‐site on DHX9 overlaid with or without bovine catalytic PKA subunit, spotted with either amino acid substitutions (B), dual N + C‐terminal truncation (C), N‐terminal truncation (D) or C‐terminal truncation (E). All peptide array figs (A–E) are representative of *N* = 2 experiments. (F) Immunoprecipitation of DHX9‐FLAG overexpressing HEK293 cells following 25 μm forskolin (FSK) + 100 μm IBMX (F/I) treatment. SDS/PAGE analysis with FLAG antibody or phospho‐(Ser/Thr) PKA substrate antibody. Quantification of ratio between phosphor‐DHX9 and DHX9^+^‐treatment, presented as mean ± SEM for *N* = 6. Statistical analysis via unpaired *t*‐test (*P = 0.049). (G) Interaction between DHX9 and phospho‐(Ser/Thr) PKA substrate in LNCaP cells alone (WT), following 10 μm rolipram (Rol) treatment (WT + Rol) or stable PDE4D7‐knockdown (P1) via proximity ligation assay (PLA). Scale bars measure 20 μm. Quantification of PLA signal between conditions are presented as mean ± SEM of *n* = 20 cells per condition for *N* = 3 independent experiments. Data normalised to WT control (at 100% as indicated by horizontal dotted line). Statistical analysis via one‐way ANOVA (ns, non‐significant, *P < 0.05). (H) Interaction between DHX9 and phospho‐(Ser/Thr) PKA substrate in LNCaPs ± 25 μm forskolin treatment via PLA. Scale bars measure 20 μm. Quantification of PLA signal for mean ± SEM of *n* = 20 cells per condition for *N* = 3 experiments. Statistical analysis via unpaired *t*‐test (****P < 0.0001). aa, amino acid; IB, immunoblot; IP, immunoprecipitation; PKA, protein kinase A.

Many cAMP signalling complexes contain a PKA substrate, the PKA holoenzyme anchored by an AKAP, and a PDE to prevent inappropriate phosphorylation of the PKA substrate motif under basal cAMP conditions [3]. We have identified a possible PKA site on DHX9 by overlaying DHX9 peptide arrays with active PKA catalytic unit then immunoblotting with a PKA phospho‐S/T substrate antibody. The site we identified contained the classical PKA motif, RRxSh (where x is any amino acid and h is a hydrophobic residue), which was identified as R^446^ R^447^ I^448^ S^449^ A^450^ in the Rec‐A like Domain 1 of DHX9 (Fig. 3A). As expected, PKA phosphorylation of the DHX9 peptide was ablated when the phospho‐accepting S^449^ was substituted with alanine, or the double arginine (R^446^ R^447^) was mutated to aspartate (Fig. 3B). The notion that the sequence R^446^ R^447^ I^448^ S^449^ A^450^ is a PKA site on DHX9 was strengthened by N‐terminal truncations that ablated the phosphorylation as soon as R^446^ was lost (Fig. 3D), or C‐terminal truncations where signal disappeared after A^450^ was removed (Fig. 3E). Simultaneous N‐ and C‐terminal truncation of the peptide also followed this trend (Fig. 3C).

Further evidence that PKA can phosphorylate DHX9 was provided by IP of DHX9 from HEK293 cells overexpressing the DHX9‐FLAG construct. Phosphorylation of FLAG‐IPs containing DHX9 could be recognised by the PKA phospho‐S/T substrate antibody, and this was significantly enhanced following treatment with forskolin + IBMX (Fig. 3F) which would dramatically increase cellular cAMP concentrations. Given the identification of the PKA‐phosphorylation site on DHX9, we decided to evaluate the occurrence of the S^449^ modification via PLA. PDE4 inhibition via rolipram treatment resulted in enhancement of the PKA phosphorylation of DHX9 suggesting the possibility that other long PDE4 isoforms in addition to PDE4D7 can also influence the PKA phosphorylation of DHX9, as the DHX9 binding region in UCR1 is conserved among isoforms. However, stable PDE4D7‐knockdown in LNCaPs led to a significant increase in phospho‐PKA substrate recognition of DHX9 (Fig. 3G). Additionally, increases in cAMP concentration in LNCaPs triggered by forskolin treatment resulted in significantly enhanced PKA‐phosphorylated DHX9 (Fig. 3H). Overall, these data indicate that DHX9 can readily be phosphorylated by PKA in LNCaP cells and enhanced via a pool of cAMP that is influenced by PDE4D7.

**Fig. 4:**
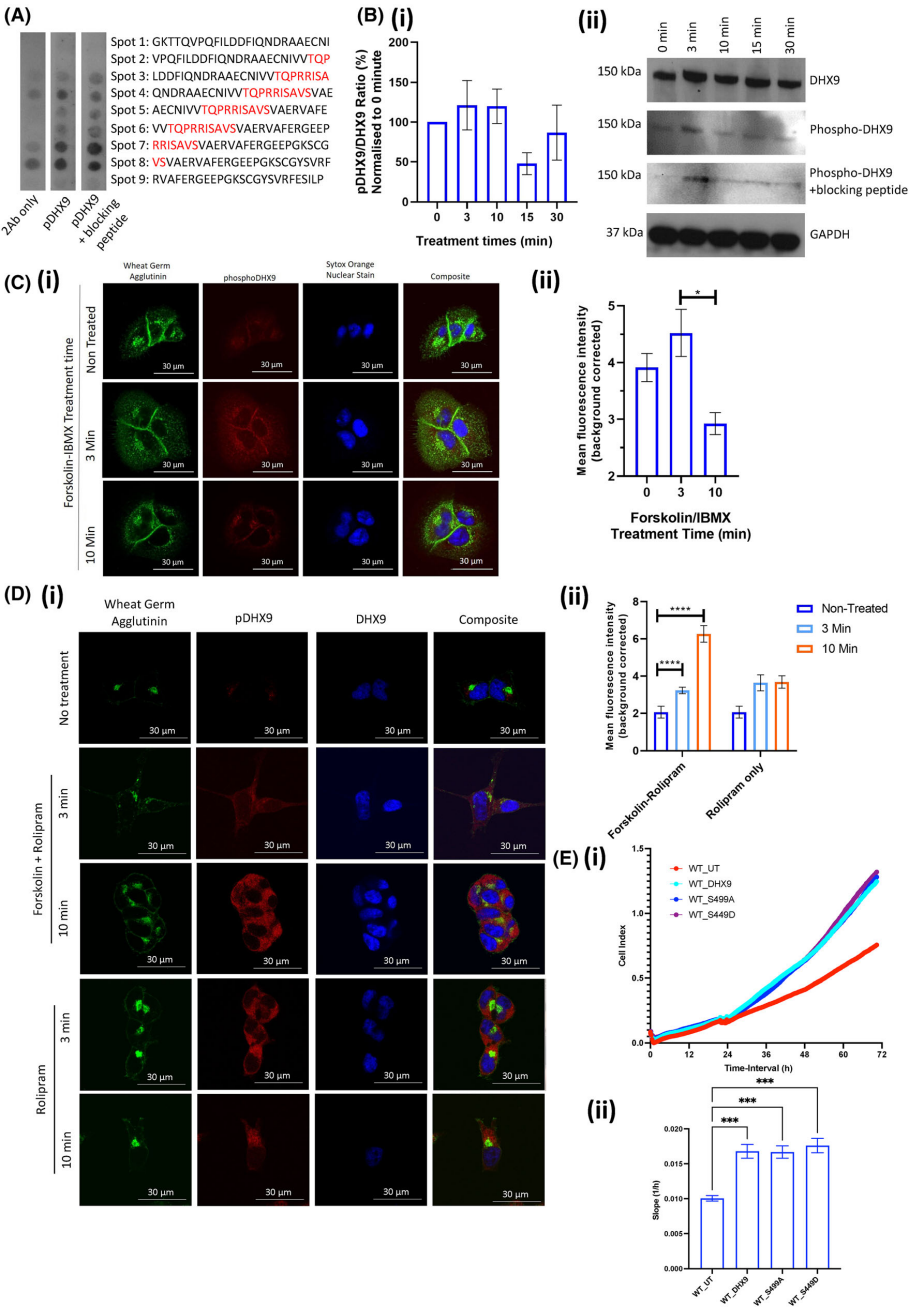
Development of phosphor‐DHX9 antibody. (A) Peptide array of phosphor‐serine 449 DHX9 sequence incubated with novel phosphor‐DHX9 (pDHX9) epitope antibody with or without DHX9‐blocking peptide or secondary antibody (2Ab) only control (*N* = 1). (B) SDS/PAGE of DHX9‐FLAG overexpressing HEK293 cells, treated with specified time course of 25 μm forskolin + 100 μm IBMX (ii) and quantified ratio of pDHX9 to total DHX9 normalised to non‐treated (0 h) control (i) (mean ± SEM for *N* = 3). No statistical significance when analysed using a one‐way ANOVA. (C) Immunocytochemistry of phosphor‐DHX9 in DU145 cells treated with specified time course of 25 μm forskolin + 100 μm IBMX (i). Scale bars measure 30 μm. Quantification of mean pDHX9 fluorescence intensity per condition (ii) as mean ± SEM for *N* = 3. Statistical analysis via one‐way ANOVA (*P = 0.0255). (D) Immunocytochemistry of phosphor‐DHX9 and DHX9 in DHX9‐FLAG and PDE4D7‐VSV overexpressing HEK293 cells following treatment with 25 μm forskolin + 10 μm rolipram (i). Scale bars measure 30 μm. Quantification of mean pDHX9 fluorescence intensity per condition (ii) as mean ± SEM for *N* = 3 experiments. Statistical analysis via mixed effects model (****P < 0.0001). (E) Real‐time growth of LNCaP cells overexpressing DHX9‐FLAG, DHX9‐S449A‐FLAG (phospho‐null DHX9 mutant) or DHX9‐S449D‐FLAG (phosphorylated DHX9 mutant) or un‐transfected (UT) analysed via the xCELLigence system (i). Slope of growth curves (mean ± SEM) measured between 0 and 72 h via simple linear regression as mean ± SEM for *N* = 3. Statistical analysis via one‐way ANOVA (***P < 0.001) (ii).

Using information from the peptide array experiments (Fig. 3) that identified the putative PKA site on DHX9 we commissioned production of a site specific S^449^ phospho‐DHX9 antibody raised against the epitope ‘TQPRRIS^p^AVS’ (where S^p^ is a phosphor‐serine). When tested on peptide array, the phospho‐DHX9 antibody detected spots that contained the phosphorylated epitope (Fig. 4A), with spot 8 showing non‐specific binding in the secondary antibody only control. We next evaluated the utility of the phospho‐DHX9 in a cellular context versus transfected FLAG‐DHX9 protein. Transfected HEK293 cells were treated with IBMX/forskolin for 30 min and a transient and significant increase in phospho‐DHX9 was detected by our antibody, which peaked at 3 min and declined to basal levels after 15 min (Fig. 4B). This corroborates data collected using the PKA phospho‐substrate antibody (Fig. 3F), suggesting that DHX9 can be phosphorylated on S^449^ in cells when cAMP levels are enhanced. Attempts to evaluate endogenous phospho‐DHX9 was done via confocal microscopy on DU145 PCa cells (Fig. 4C) which express very low levels of PDE4D7 (Fig. S1). Phosphorylated DHX9 could be detected at basal levels presumably as PDE4D7 is deficient, and this was increased by the addition of forskolin/IBMX, peaking at 3 min. Interestingly, endogenous phosphorylated DHX9 could be seen at the peri‐nuclear and membranes of the cells but not within the nucleus suggesting that PKA phosphorylation may influence the cellular location of the helicase.

To determine whether rolipram (PDE4 specific inhibitor) could increase phospho‐DHX9 levels, transfected HEK293 cells were treated with a rolipram or forskolin/rolipram time‐course and phospho‐DHX9 levels evaluated. Rolipram enhanced cytoplasmic phospho‐DHX9 levels at 3‐ and 10‐min treatment which suggests a role for PDE4 under basal cAMP conditions. Forskolin/rolipram treatment produced an expected larger and more sustained response that was significantly enhanced over basal levels (Fig. 4D).

DHX9‐FLAG phospho‐mimic (S449D) and phospho‐null (S449A) mutants were created via site‐directed mutagenesis of S^449^ at the putative PKA phosphorylation site of DHX9 (Fig. S4). Overexpression of DHX9 in LNCaP cells significantly induced upregulation of cell growth (Fig. 4E), an effect also observed in HEK293 cells (Fig. S5), revealing that increased DHX9 expression exerts pro‐proliferative effects not limited to PCa cells. Transient transfection of S449D or S449A DHX9 mutants also increased cell proliferation to the same extent as WT DHX9, suggesting that phosphorylation of DHX9 at S449 does not affect LNCaP growth characteristics (Fig. 4E).

### 3.4 Effect of PDE4D7 on DHX9 localisation and disruption of PDE4D7‐DHX9 interaction on PCa phenotype

**Fig. 5:**
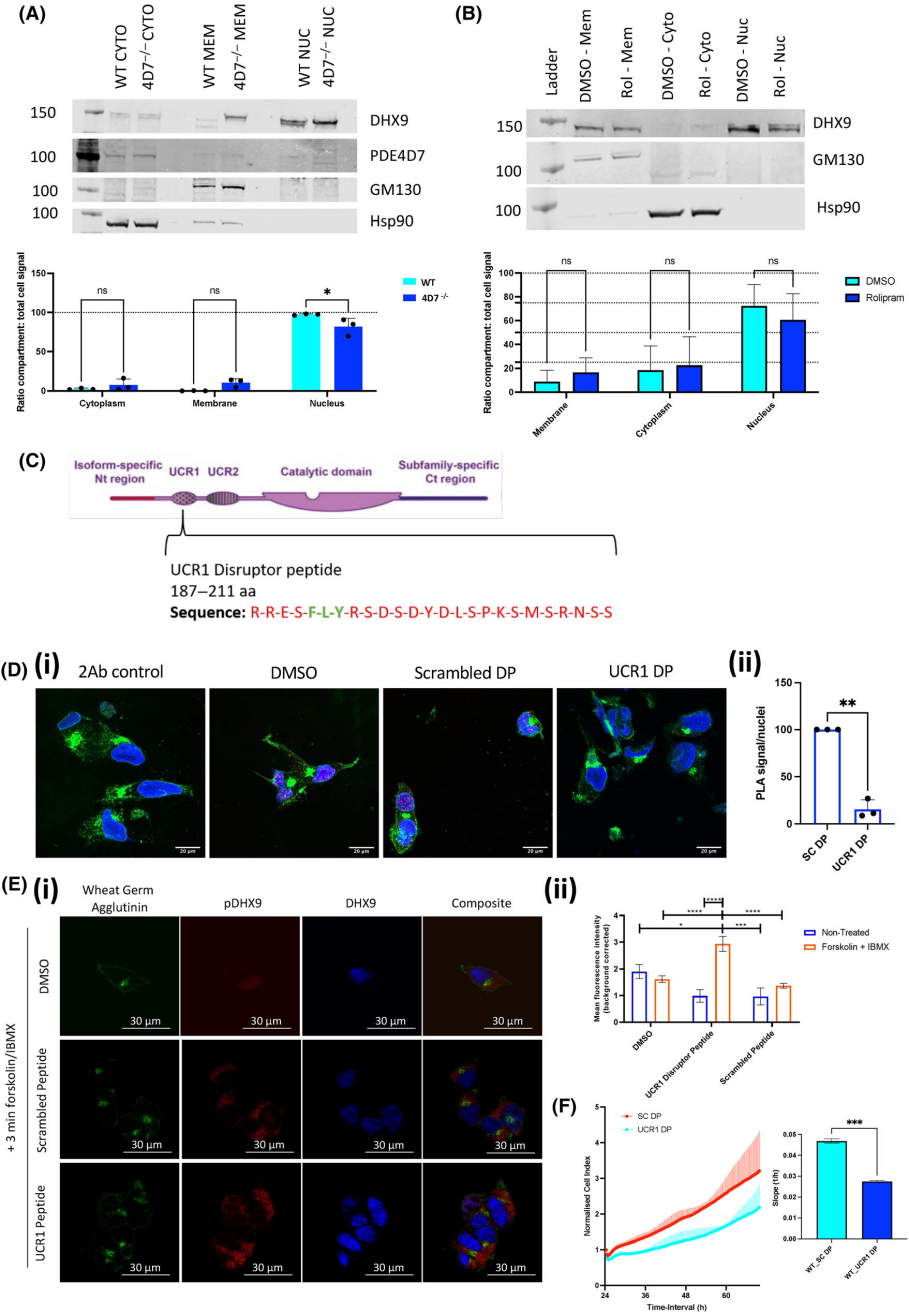
Disruption of PDE4D7‐DHX9 complex in prostate cancer cells. (A, B) Subcellular fractionation of wild‐type (WT) or stable PDE4D7‐knockdown (P1) LNCaP cells (A) or LNCaP cells with 10 μm rolipram treatment or 0.1% DMSO (B). SDS/PAGE analysis of PDE4D7 and DHX9 protein, alongside membrane (GM130) and cytosol (Hsp90) markers. Quantification of DHX9 expression per compartment compared to total cell signal as mean ± SEM for *N* = 3. Statistical analysis via Two‐way ANOVA (*P < 0.05, ns, non‐significant). (C) Design of cell permeable disrupting peptide targeting DHX9 binding region within UCR1 domain of PDE4D7. Protein structure diagram adapted from Tibbo et al. [50] under the CCBY 4.0 licence. (D) Disruption of endogenous PDE4D7 and DHX9 interaction via novel UCR1 disruptor peptide analysed via proximity ligation assay (PLA) in LNCaP cells. Cells were treated with either 0.1% DMSO, scrambled peptide (scrambled DP) or UCR1 disruptor peptide (UCR1 DP). Scale bars measure 20 μm. Quantification of PLA signal for each treatment condition normalised to DMSO treatment, presented as mean ± SEM for *N* = 3 independent experiments. Statistical analysis via one sample *t*‐test (**P ≤ 0.01). (E) Immunocytochemistry of pDHX9 and DHX9 expression in DHX9‐FLAG and PDE4D7‐VSV overexpressing HEK293 cells treated with 0.1% DMSO, scrambled DP or UCR1 DP, following 25 μm forskolin + 100 μm IBMX treatment. Scale bars measure 30 μm. Quantification of mean pDHX9 fluorescence intensity with or without forskolin/IBMX treatment, presented as mean ± SEM for *N* = 3 independent experiments. Statistical analysis via mixed effects model (*P = 0.0138, ***P = 0.0007, ****P < 0.0001). (F) Real‐time growth analysis of LNCaP cells via the xCELLigence system following treatment with UCR1 DP or scrambled peptide (SC DP). Normalised cell index to timepoint of treatment. Quantification via slope of normalised growth curves was measured between 0 and 48 h post‐treatment via simple linear regression. Data presented as mean ± SEM for *N* = 3 independent experiments. Statistical analysis via unpaired *t*‐test (***P < 0.001). 2Ab, secondary antibody; aa, amino acid; Ct, C‐terminal; CYTO, cytoplasm; MEM, membrane; Nt, N‐terminal; NUC, nucleus; ROL, rolipram; UCR1/2, upstream conserved region 1/2.

Given the robust indication of an interaction between PDE4D7 and DHX9 in LNCaP cells (Fig. 1E), and evidence suggesting that PKA phosphorylation may influence DHX9 localisation (Fig. 4C), we investigated whether reduction of this interaction via PDE4D7‐knockdown could affect DHX9 localisation. Subcellular fractionation and western blotting revealed significant translocation of DHX9 from the nucleus of PDE4D7‐knockdown LNCaP cells (P1) in comparison to WT, with corresponding increases of DHX9 observed in the membrane and cytoplasm (Fig. 5A). This indicates that loss of PDE4D7 and thereby the interaction between PDE4D7‐DHX9 leads to altered localisation of DHX9 within the cell. A similar movement of DHX9 out of the nucleus could also be triggered by the PDE4‐specific inhibitor, rolipram (Fig. 5B), suggesting that the new distribution of DHX9 was PDE4‐activity dependent. As Rolipram inhibits concomitantly all 25 PDE4 isoforms with a similar affinity, we decided to develop a cell‐penetrating peptide to disrupt the PDE4D7‐DHX9 interaction (Fig. 5C) as this would allow a more specific method to remove the localised PDE4 activity from the vicinity of DHX9 [10]. Using information from peptide arrays (Fig. 2), we selected the peptide encompassing R^187^ – S^212^ within the PDE4D7 UCR1 region which was a robust binder of DHX9. The peptide was rendered cell permeable by virtue of the addition of a stearate group as previously used by us on other occasions in the design of PDE4 disruptors [25, 26, 27]. In addition, a control scrambled peptide was produced as a negative control. Successful disruption of the PDE4D7‐DHX9 interaction following peptide administration was confirmed using PLA, with significant downregulation of PLA signal observed between scrambled peptide and UCR1 disruptor peptide (DP) treated LNCaPs (Fig. 5D).

Displacement of PDE4D7 from DHX9 via UCR1 disruptor peptide, prior to upregulation of cAMP levels via forskolin and IBMX treatment, led to a significant increase in phopho‐DHX9 levels in comparison to the scrambled peptide and DMSO‐treated controls (Fig. 5E). Additionally, it can be observed that cellular localisation of total DHX9 and phosphor‐DHX9 shifts, with phosphorylation promoting nuclear to cytoplasmic translocation (Fig. 5E). To ascertain the functional implications of the disruption of the PDE4D7‐DHX9 complex in PCa we analysed real‐time growth of LNCaP cells following treatment with the peptide disruptor. Disruption of the PDE4D7‐DHX9 complex resulted in a significant reduction in growth of LNCaP cells (Fig. 5F) when compared to the scrambled control.

## 4 Discussion

### 4.1 Interaction between PDE4D7 and DHX9

Regulation of intracellular signal transduction is tightly coordinated both directly and indirectly by PPIs. With regards to cAMP signalling, spatiotemporal regulation via PDE4s shapes localised cAMP gradients which, in turn, activate a localised set of cAMP effector proteins to effect cell physiology. PDE4 localisation is therefore a major determinant of the enzyme's many functions; however, this relies on the anchoring of specific PDE4 isoforms to certain signalling complexes via protein–protein interactions [28]. A prime example of PDE4‐protein interactions is the PDE4‐β‐arrestin complex, whereby activation of the β2 adrenoreceptor (β2AR) recruits β‐arrestin bound to PDE4 (with a preference for PDE4D5), leading to cAMP hydrolysis and regulation of PKA driven desensitisation of β2AR activity [29]. There are many other PDE4 PPIs, including PDE4A4/5–P75 NTR and the PDE4D5–Integrin α5 complex, as reviewed by Blair and Baillie [30].

Much work has positioned PDE4D7 as a prognostic biomarker for prostate cancer due to its expression inversely correlating with disease progression and recurrence [5, 31]. Akin to this, DHX9 expression has also been linked to numerous cancers, such as colorectal, lung and breast cancer [32, 33] and more recently prostate cancer [20]. Here, for the first time, we report identification of a protein complex between PDE4D7 and the RNA/DNA helicase DHX9.

Our data suggests that the complex is maintained by a docking motif for DHX9 on the UCR1 domain of PDE4D7, specifically at the conserved multi‐functional ‘FLY’ binding site essential for several protein interactions [24]. As this motif is conserved between all PDE4 long forms, there is the potential that DHX9 can also bind to other PDE4s, however given that PDE4D7 shows the most significant expression among PDE4 isoforms in PCa [5], it can be assumed that the majority of PDE4‐linked DHX9 is bound to PDE4D7. The interaction of PDE4D7 with DHX9 is mediated through DHX9's Rec‐A like domain 2 within the helicase core domain. Other known DHX9 interactors, such as Nup98, have been shown to regulate DHX9 helicase activity [34], therefore it could be postulated that the interaction with PDE4D7 at the helicase domain may also influence activity. In short, our data suggests that PDE4D7 and DHX9 exist as part of a signalosome with a fundamental role in PCa progression, such as modulating DHX9 helicase activity and downstream signalling pathways implicated in PCa.

Another interesting observation is that disruption of the PDE4D7‐DHX9 interaction through PDE4D7‐knockdown or PDE4 inhibition alters the cellular localisation of DHX9. Upon PDE4D7 silencing in PCa cells a significant percentage of DHX9 shuttles out of the nucleus and into the cytosol and membrane compartments, with a similar trend observed upon total PDE4 inhibition via rolipram. This indicates that expression of PDE4, in particular PDE4D7, plays a role in the maintenance of DHX9's occupation within the nucleus where it is able to regulate processes crucial to maintenance of genomic stability, such as in DNA damage repair, transcription and DNA replication [15]. Whilst PDE4D7 is primarily membrane/cytosolic in distribution, DHX9 is predominantly expressed in the nucleus, however due to the presence of NES and NLS sequences it is able to shuttle into the cytoplasm to regulate cellular processes such as translation and miRNA processing [32]. Here, DHX9 also functions as a cytosolic RNA and DNA sensor in the innate immune response against viral infection [35, 36], which can be exploited for enhancing oncolytic viral therapy [37]. DHX9 cytosolic localisation also occurs during mitosis and upon inhibition of transcription [38]. In light of this, it could be speculated that enhanced occurrence of DHX9 in the cytoplasm depends on inhibited transcription of PDE4(D7). Also, as this altered localisation occurs during mitosis, upregulated mitosis (enhanced proliferation) upon PDE4D7‐downregulation in PCa [5] may result in altered DHX9 localisation. Interestingly, DHX9 and the androgen receptor (AR) are both able to localise within the cytoplasm and nucleus. Chellini et al. [20] found that the AR binds to the DHX9 promoter in LNCaP cells, and depletion of DHX9 not only hinders growth of PCa cells but also alters transcription of many AR‐target genes, suggesting that the AR‐DHX9 axis is an important feature of PCa development. Whilst PDE4D7 and DHX9 have predominantly different subcellular locations, previous research has shown that proteins with even a slightly overlapping distributions form functional complexes, such as PDE4 and POPDC, and the physiological function uncovered via a disruptor peptide [25].

Our results suggest that PDE4D7 modulates cAMP gradients around DHX9 in PCa. In late‐stage PCa cells (as reflected by a low PDE4D7 expression [5]) a higher percentage of DHX9 shuttles into the cytosol, impacting DHX9's diverse range of cellular functions.

### 4.2 PKA phosphorylation site on DHX9 mediated by PDE4D7

In addition to the interaction between DHX9 and PDE4D7, we also report a novel PKA phosphorylation site on DHX9. As previously discussed, cAMP signalling is crucial for the strict coordination of intracellular signalling cascades through PKA‐mediated phosphorylation of intermediates to regulate key processes such as proliferation, survival, and repair [28]. Regarding DHX9, its phosphorylation promotes therapy resistance in leukaemia and colorectal cancer cells [39, 40]. Whilst DHX9 is known to be phosphorylated by DNA‐dependent protein kinase (DNA‐PK) and phosphatidylinositol 3‐kinase‐related kinases (PI3KKs) [40, 41], this is the first study to show that DHX9 is a putative substrate for PKA. Peptide array identified a single serine at residue 449 within the helicase domain of DHX9 can be phosphorylated. This amino acid is located upstream of the PDE4D7 binding site on DHX9, and loss of this serine prevented DHX9 phosphorylation. Similarly, PDEs are PKA substrates whereby phosphorylation within their UCR1 domain leads to their activation [42], however PDE4D7 is also able be phosphorylated by PKA at a unique site within its N‐terminus leading to a reduction in its activity [43].

Upon mapping the PKA motif on DHX9, we developed a custom phospho‐DHX9 antibody to further assess the DHX9 phosphorylation status in PCa cells. Unsurprisingly, we found that PDE4D7 negatively regulates DHX9 phosphorylation. Increasing cAMP levels via treatment with forskolin and rolipram led to a significant increase in phosphorylation of DHX9. Similarly, 3‐min treatment with forskolin and IBMX enhanced phospho‐DHX9 levels in HEK293 and DU145 cells. Decreased PDE4D7 expression observed in late‐stage PCa contributes to elevated cAMP levels and activation of PKA, likely to result in enhanced DHX9 phosphorylation. Due to the interaction between PDE4D7 and DHX9, it appears that PDE4D7 can hydrolyse pools of cAMP localised around DHX9, thereby inhibiting its PKA‐mediated phosphorylation. Ultimately, this could potentially regulate DHX9's helicase activity or cellular distribution, contributing to PCa progression. To further assess DHX9 phosphorylation in PCa we developed phospho‐null (S449A) and phospho‐mimic (S449D) mutants of DHX9 and assessed the functional implications of DHX9 overexpression in PCa. Given that depletion of DHX9 is known to hinder PCa growth [20], it is unsurprising that transient DHX9 overexpression significantly enhanced proliferation. This effect was also observed by with stable lentiviral‐mediated DHX9 overexpression, which significantly enhanced proliferation in a range of colorectal cancer cell lines [44]. Interestingly, WT DHX9 and phosphor‐mimic mutants all produced a similar effect on cell growth, however it is questionable how physiological transient transfection of DHX9 is and it is impossible to know whether the S to D substitution is really mimicking the phosphorylation. On this note, Paleologou et al. [45] examined the phosphorylation‐mimicking mutants against WT and purified phosphorylated Ser(P)‐129 α‐syn, and found that the phosphor‐mimics (S129E/D) did not reproduce the effects of phosphorylation at Ser129 *in vitro*, highlighting that phospho‐site substitutions are not always a reliable model of physiological events. Future research should assess how PKA‐mediated DHX9 phosphorylation can alter its helicase activity and its other functions.

### 4.3 Disruption of PDE4D7‐DHX9 interaction

#### 4.3.1 PDE4D7‐DHX9 interaction is crucial for PCa growth

As previously discussed, many PPIs are relevant in diseases including cancer. Cell‐penetrating peptides (CPPs) offer many therapeutic advantages over other anti‐cancer compounds due to their specificity, rapid delivery into tumours and low toxicity, as well as being relatively easy to synthesise [46]. Disruptor CPPs targeting PDE‐protein interactions can provide an effective and specific method of PDE4 inhibition that depends on displacement rather than targeting the active site. This means that only a small percentage of the total pool of the PDE is affected, lessening the chances of ‘off‐target’ consequences [10, 28].

An example of the use of therapeutic peptides targeting PDE‐protein interactions is between PDE8A and C‐Raf. Following on from the discovery of an interaction between PDE8A and C‐Raf in melanoma which enhances its progression [27], Blair et al. [47] mapped the PDE8A‐C‐Raf binding domain, allowing generation of a cell‐permeable disruptor peptide targeting this interaction which hindered growth of melanoma cells, suggesting its potential as a treatment for B‐Raf inhibitor resistant melanoma. Given the interaction identified between PDE4D7 and DHX9, we wanted to determine what effect disrupting this complex has in PCa cells. We designed a CPP against the DHX9‐PDE4D7 complex and PLA confirmed that the CPP significantly inhibited complex formation between PDE4D7 and DHX9 in LNCaP cells. Displacement of PDE4D7 from DHX9 also conferred decreased cell growth in LNCaPs, mirroring the effect observed from the PDE8A‐C‐Raf CPP in melanoma cells, further postulating the potential of targeting PDE‐protein interactions as a novel therapy for cancers [47].

#### 4.3.2 Displacement of PDE4D7‐DHX9 alters DHX9 phosphorylation

By targeting specific PDE‐protein interactions, regulation of cAMP signalling at precise intracellular nanodomains can be established [30]. Disruption of the PDE4D7‐DHX9 interaction via our CPP led to an increase in PKA phosphorylation of DHX9, highlighting the complex's importance in modulating this post‐translational modification. Upregulation of cAMP due to PDE4D7 down regulation in AI/CRPC cells would theoretically leave DHX9 unprotected against PKA phosphorylation. Akin to this, another PDE4D interacting protein, HSP20, can be phosphorylated by PKA in order to confer a cardio‐protective mechanism and a CPP disrupting the Hsp20‐PDE4D complex similarly led to enhanced PKA‐mediated phosphorylation [48, 49].

## 5 Conclusion

Here, we have identified for the first time a PCa‐relevant, cAMP signalosome containing PDE4D7 and DHX9 that influences cancer growth. Mechanistically, we hypothesise that PDE4D7 controls both the cellular location and PKA‐phosphorylation status of the helicase, maintaining it in a predominantly nuclear, unphosphorylated state. Displacing the PDE4D7 complex results in hindered PCa growth, positioning the PDE4D7‐DHX9 complex as a potential novel therapeutic target for the disease, however further research on the effects of this interaction and its displacement is required to further ascertain this.

## Conflict of interest

RH is an employee of Philips Research. GSB and RH hold patent rights related to PDE4D7 and DHX9. The authors declare no conflict of interest.

## Author contributions

CG, TB, AB and JEF undertook the work and analysed the data. CG and GSB wrote the manuscript. GSB and RH contributed to supervision and manuscript revision. All authors have read and approved this manuscript.

### Peer review

The peer review history for this article is available at https://www.webofscience.com/api/gateway/wos/peer‐review/10.1002/1878‐0261.13572.

## Supporting information


**Fig. S1.** PDE4D isoform and DHX9 expression between DU145, LNCaP and VCaP prostate cancer cells.
**Fig. S2.** PDE4D7 expression upon enzalutamide treatment.
**Fig. S3.** PDE4D UCR1‐GST purification from BL21 *E. coli*.
**Fig. S4.** Site‐directed mutagenesis of DHX9 with point mutations at Ser449.
**Fig. S5.** DHX9 overexpression in HEK293 cells.

## Data Availability

The datasets used and/or analysed during the current study are available from the corresponding author on reasonable request.
