# Supplementary material for: cAMP‐phosphodiesterase 4D7 (PDE4D7) forms a cAMP signalosome complex with DHX9 and is implicated in prostate cancer progression
Source: Mol Oncol. 2024 Jan 5;18(3):707–25. doi: 10.1002/1878-0261.13572 (PMC10920091; doi:10.1002/1878-0261.13572)
Supplement: Supplementary file 1 — Fig. S1. PDE4D isoform and DHX9 expression between DU145, LNCaP and VCaP prostate cancer cells. Fig. S2. PDE4D7 expression upon enzalutamide treatment. Fig. S3. PDE4D UCR1‐GST purification from BL21 E. coli. Fig. S4. Site‐directed mutagenesis of DHX9 with point mutations at Ser449. Fig. S5. DHX9 overexpression in HEK293 cells. [file MOL2-18-707-s001.docx]

# Supplementary Figures

***Sup Fig 1: PDE4D isoform and DHX9 expression between DU145, LNCaP and VCaP prostate cancer cells.*** *A)* *RT-qPCR of PDE4D5/7/9 and DHX9 expression. Fold changes in gene expression are shown relative to DU145 (2^-∆∆Ct^) for mean ± SEM for N=3. Statistical analysis via one-way ANOVA on ∆Ct values. B) SDS-PAGE analysis of PDE4D isoform and DHX9 protein expression. PDE4D7-VSV overexpressing HEK293 cells were used as a positive control. Quantification via densitometry normalised to DU145 cells, representing mean ± SEM for N=3. Statistical analysis via one sample t-test values (* p ≤ 0.05, ** p ≤ 0.01, *** p ≤ 0.001, **** p ≤ 0.0001, ns = non-significant).*

***Sup Fig 2: PDE4D7 expression upon enzalutamide treatment.*** *A) SDS-PAGE analysis of PDE4D7 expression in LNCaP cells treated with 10 µM enzalutamide (ENZ) or 0.1% DMSO. PDE4D7-VSV overexpressing HEK293 cells were used as a positive control. Quantification via densitometry normalised to DMSO (N=3, mean ± SEM). Statistical analysis via one sample t-test did not reveal any significant difference.*

***Sup Fig 3:*** ***PDE4D UCR1-GST purification from BL21 E. coli.*** *UCR1-GST and GST purified protein analysed via SDS-PAGE and incubated with GST antibody.*

***Sup Fig 4:*** ***Site directed mutagenesis of DHX9 with point mutations at Ser449.*** *Confirmation of point mutations in comparison with human DHX9 via NCBI BLAST for S449A (A) and S449D (B). HEK293 cells were transfected with DHX9-S449A-FLAG (C) or DHX9-S449D-FLAG (D). SDS-PAGE confirmation of transfection and expected size of bands relative to WT DHX9-FLAG. Un-transfected HEK293 served as negative control.*

***Sup Fig 5: DHX9 overexpression in HEK293 cells.*** *A) Real-time growth of un-transfected (UT) or DHX9 plasmid transfected (DHX9) HEK293 (HEK) cells analysed via xCELLigence. B) Slope of growth curves measured via simple linear regression (mean ± SEM) between 0-72h for N=3. Statistical analysis via unpaired t-test (*** p < 0.001).*
